# Late Onset Thrombotic Microangiopathy in Kidney Transplants; Poor Outcome Despite Eculizumab Treatment

**DOI:** 10.3389/ti.2025.15404

**Published:** 2025-11-24

**Authors:** Emily K. Glover, Emma K. Montgomery, Edwin K. S. Wong, Sally Johnson, Michal Malina, Kevin J. Marchbank, David Kavanagh, Neil S. Sheerin

**Affiliations:** 1 National Renal Complement Therapeutics Centre, Newcastle upon Tyne Hospitals NHS Foundation Trust, Newcastle upon Tyne, United Kingdom; 2 Translational and Clinical Research Institute, Newcastle University, Newcastle upon Tyne, United Kingdom; 3 Great North Children’s Hospital, Newcastle Upon Tyne Hospitals NHS Foundation Trust, Newcastle upon Tyne, United Kingdom

**Keywords:** kidney, transplantation, rare disease, recurrence, thrombotic microangiopathy, aHUS, eculizumab

## Abstract

Atypical hemolytic uremic syndrome (aHUS) is a rare cause of end stage kidney disease (ESKD) associated with a high rate of recurrence in kidney transplants causing a post-transplant thrombotic microangiopathy (TMA). Prophylactic eculizumab can prevent disease recurrence in select patients. Treating at the time of post-transplant TMA occurrence is the only option if the diagnosis of aHUS is not established pre-transplant. We report our experience of using eculizumab at the point of post-transplant TMA in those with a diagnosis or suspicion of aHUS. We conducted a case note review of 26 patients treated with eculizumab for post-transplant TMA. Screening for complement pathway defects included testing for variants in genes of the complement pathway and anti-factor H autoantibodies. 34.6% of recipients had an identified complement pathway defect. Median time to presentation with post-transplant TMA was 8.4 months. Death-censored graft survival 12 months after starting eculizumab was 68% for the cohort and was worse in those presenting >12 months post-transplant where this figure was 42.9%. The outcome is poor despite eculizumab treatment for those presenting >12 months after transplantation with TMA.

## Graphical Abstract

**Figure:**
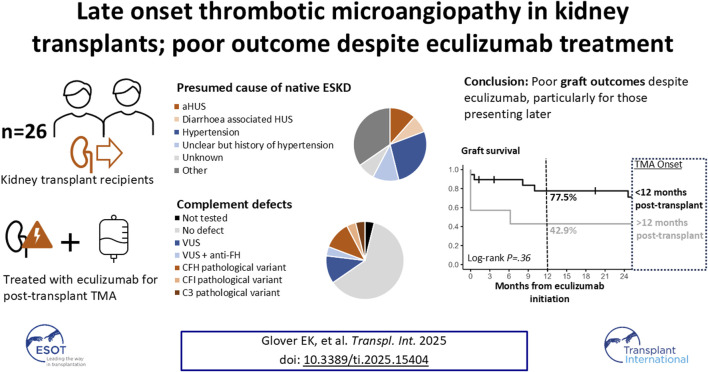

## Introduction

Atypical hemolytic uremic syndrome (aHUS) is a rare cause of thrombotic microangiopathy (TMA) with a high rate of recurrence in kidney transplantation, particularly in the early post-transplant period [1–3]. It is a complement mediated disease that can be treated with eculizumab, a monoclonal antibody targeting C5 to block complement terminal pathway activity [4–7]. Prophylactic eculizumab treatment, starting from transplantation, is effective at reducing aHUS recurrence and graft loss in certain individuals with medium and high risk complement pathway defects (Supplementary Table S1) [1, 8, 9]. However, this approach is not available to those without an existing diagnosis of aHUS, presenting for the first time with TMA after transplantation.

Diagnosing complement mediated aHUS as the cause of post-transplant TMA is challenging, as it cannot be readily distinguished on biopsy from other causes of TMA including calcineurin inhibitor (CNI) toxicity, antibody mediated rejection (AMR), anti-phospholipid syndrome, ischemia reperfusion injury, bleeding, viral infections, recurrence of primary disease (systemic lupus erythematosus, scleroderma) and Shiga toxin-producing *Escherichia coli* HUS [10, 11]. Genetic testing and factor H (FH) autoantibody testing for causes of alternative complement pathway dysregulation are key in supporting a diagnosis of aHUS. However, for those presenting *de novo* post-transplant a provisional diagnosis will be required whilst awaiting these results.

In those with an existing diagnosis of aHUS, data from Zuber et al. [8, 12] has shown a benefit in treating aHUS recurrence at the point of post-transplant TMA occurrence with eculizumab. In the larger of their cohorts, all recipients also had a high or medium risk complement pathway defect [8]. Duineveld et al. have found reactive eculizumab treatment at the point of aHUS recurrence to be a reasonable approach, particularly when coupled with a strategy to minimise endothelial injury [13, 14].

In England, patients with suspected aHUS are referred to a single specialist centre [15] for further investigation and access to eculizumab. We report the features and outcomes of post-transplant TMA in kidney transplant recipients eligible for eculizumab treatment for a suspected diagnosis of aHUS.

## Patients and Methods

### Study Group

We conducted a retrospective case note review of patients referred to the National Renal Complement Therapeutic Centre, Newcastle upon Tyne, UK (NRCTC; http://www.atypicalhus.co.uk) for post-transplant TMA and who were treated with eculizumab for this episode. Patients who received prophylactic eculizumab treatment from implantation of their kidney transplant were excluded.

Referrals made before 14th March 2021 were included to allow sufficient follow-up after eculizumab initiation.

The decision to treat with eculizumab post-transplant for TMA occurrence was made through the NRCTC multidisciplinary team meeting and considered timing of presentation in the post-transplant period, hematological evidence of microangiopathic hemolytic anaemia (MAHA), thrombocytopenia, transplant biopsy results and cause of native end stage kidney disease (ESKD). Complement pathway abnormality screening was requested on all referrals but results were frequently not available at the time of decision making. Only those with a known diagnosis of aHUS, HUS or an unconfirmed cause of native ESKD would be considered for eculizumab to treat post-transplant TMA. In recipients where the native kidney disease had been attributed to hypertension, there was a higher suspicion of complement mediated disease as a high prevalence of complement pathway defects has been identified in this group [11, 16]. Other causes of post-transplant TMA had to be considered unlikely to approve eculizumab, with particular considerations for exclusion being concerns of CNI toxicity, histological evidence of AMR and a clear non-complement mediated native kidney disease diagnosis. In all cases, other causes of TMA were considered depending on the clinical scenario.

The dosing schedule for adults starting eculizumab was 4 weekly doses of 900 mg, one dose of 1,200 mg after a further week, then 1,200 mg every 2 weeks [17]. Dose adjustments for children and in the case of significant blood loss or breakthrough complement activity have been previously described [1, 17].

To protect against the increased risk of *Neisseria meningitides* infection whilst on eculizumab [17], vaccination against serotypes ACWY and B was required before starting eculizumab or within 2 weeks of starting [18]. Prophylactic antibiotics were also recommended whilst treatment continued [18].

### Data Collection

Available medical notes were reviewed for transplant recipient sex, age at transplantation with current transplant, year of transplantation, time post-transplant of eculizumab initiation, reason for eculizumab cessation with a functioning graft, graft failure defined as chronic dialysis or re-transplant, cause of death in those who died with a functioning graft, cause of native ESKD and cause of previous transplant loss. Where available, data on native and transplant kidney biopsy results, transplant mismatch, ABO incompatibility, presence of DSAs, immunosuppressive regime and patient survival after graft loss were collected. Any previous use of eculizumab was recorded.

For the purposes of this work, *de novo* disease was defined as being when the first recognition of TMA thought secondary to aHUS occurred post-transplant and so a diagnosis of aHUS was not present at the time of implantation.

### Screening for Complement Pathway Defects

Variant screening in complement pathway genes *CFI,* [19] *CFH,* [20] *CFB,* [21] *C3* [22] and *CD46* [23] and chromosomal rearrangements [24, 25] *(CFH, CFRH1, CFHR2, CFHR3, CFHR4, CFHR5, CD46* and *CFI)* and in non-complement genes associate with aHUS (*DGKE*, [26] *MMACHC*, [1]*VTN*, [1] *PLG*, [27] *THBD*, [1] *IFN2* [28]) was conducted as previously described.

Rare genetic variants were evaluated using Alamut Visual 2.10 (2017 Interactive Biosoftware). Variants were classified in 2019 according to American College of Medical Genetics and Genomics guidelines with refinement developed by Sequence Variant Interpretation Working Group.

Anti-FH autoantibody testing was performed in selected cases using the consensus ELISA assay, as previously described [1, 29].

### Statistics

Kidney graft survival was analysed with Kaplan-Meier curves and censored for patient death with a functioning graft and for functioning graft at last follow. Log-rank test assessed the difference between survival of groups.

Subgroup characteristics were compared with Fisher exact test for categorical variables, t-test to compare means of numerical variables and Kruskal-Wallis to compare medians of time to presentation.

Analysis was performed using Rstudio Team (2021). RStudio: Integrated Development Environment for R. R Studio, PBC, Boston, MA URL http://www.rstudio.com/. *P* < 0.05 was considered statistically significant.

## Results

### Demographics

Our cohort consists of 26 transplant recipients treated with eculizumab for post-transplant TMA (Supplementary dataset). The majority (73.1%) of transplant recipients were female. There was one child (#4) included in the cohort, age nine at both transplantation and eculizumab initiation.

Transplants were implanted between 2005 and 2020 (median 2016) with referrals for post-transplant TMA being made between 2012 and 2021.

### Previous Renal History

#### Cause of ESKD and Native Kidney Biopsies

Causes of native ESKD and biopsy results are given in Table 1 and included three with recognised aHUS before transplantation. All recipients with evidence of TMA (n = 8) or microangiopathy without thrombosis (n = 1) on native kidney biopsy had an ESKD diagnosis of aHUS, HUS or a history of hypertension. Rationale for eculizumab treatment in those with alternative initial native kidney diagnoses is detailed in Table 1, with many in this group having no native kidney biopsy result.

**TABLE 1:** Native kidney disease history of recipients (n = 26) treated with eculizumab for post-transplant thrombotic microangiopathy (TMA) detailed as pre-transplant diagnosis for end stage kidney disease (ESKD) and associated native renal biopsy findings.

Cause of ESKD	Native renal biopsy	Rationale for eculizumab treatment
Existing diagnosis of aHUS		
aHUS (n = 3)	TMA (n = 3)	
*De novo*		
Hypertension (n = 7)	TMA (n = 2) Consistent with hypertension (n = 2) Extensive chronic damage (n = 1) Microangiopathy (no thrombosis) (n = 1) Biopsy not available (n = 1)	Involvement of hypertension in native kidney disease
Unclear cause but history included hypertension (n = 3)	Extensively damaged parenchyma with possibility of TMA (n = 1) TMA (n = 1) Biopsy not available (n = 1)	
Diarrhoea associated HUS (n = 2)	TMA (n = 1) Biopsy not available (n = 1)	Previous diarrhoea associated HUS.
Unknown (n = 2)	Fibrosis (n = 1) Biopsy not available (n = 1)	Unclear native kidney diagnosis
Reflux nephropathy (n = 2)	Biopsy not available (n = 1) Data not available (n = 1)	Previous graft loss from TMA at 5 years in one and family history of possible aHUS in the other
Chronic pyelonephritis (n = 1)	Biopsy not available (n = 1)	Uncertainty surrounding native kidney disease as presented with two small kidneys
HIV nephropathy (n = 1)	Biopsy not available (n = 1)	Pathological *CFI* variant and previous early graft loss from arterial thrombus and lack of native kidney biopsy
FSGS (n = 2)	FSGS (n = 2)	FSGS considered secondary with unclear cause in one case and following diarrhoea associated HUS in childhood for the other
IgA nephropathy (n = 1)	Crescentic IgA (n = 1)	Previous graft with TMA on biopsy at 8 months and graft loss at 19 months
Mesangial proliferation (n = 1)	MPGN, later reclassified as mesangial proliferation (n = 1)	Native kidney disease was initially described as MPGN on biopsy which can be complement mediated
Bilateral nephrectomy (n = 1)	Wilm’s tumour (n = 1)	Highly sensitized patient unlikely to be retransplanted and with *CFI* variant of uncertain significance

Reasons for considering complement mediated disease, and therefore rationale for eculizumab treatment, in those without an existing diagnosis of atypical hemolytic uremic syndrome (aHUS) before this transplant was implanted are detailed. FSGS, focal segmental glomerulosclerosis; HUS, hemolytic uremic syndrome; MPGN, membranoproliferative glomerulonephritis.

#### Previous Causes of Graft Loss

Nine recipients included in our cohort had previously lost a kidney transplant including two from post-transplant TMA and a further showing IgA recurrence on biopsy with concurrent TMA. One recipient had lost two previous transplants with one attributed to rejection and the other suspected hydronephrosis or CMV. The remaining five previous transplants failed from arterial thrombus, primary non function, AMR (n = 2) and chronic allograft nephropathy.

No transplant recipients included in our cohort had received eculizumab before.

### Complement Defect Status

Identified complement defects are detailed in Table 2. The majority of those tested (n = 25) had no identified complement pathway abnormality (64%). One recipient died shortly after presentation and so was not tested. One of those tested (n = 20) had anti-FH autoantibodies detected and this was alongside a variant of uncertain significance (VUS).

**TABLE 2:** Complement pathway abnormalities detected in transplant recipients tested for genetic variants (n = 25) and autoantibodies against factor H (anti-FH; n = 20).

Genetic testing	N (% tested)
No defect identified (%)	16 (64)
Variant of Uncertain Significance (%)	4 (16)
Pathological variant (%)	5 (20)
*CFH*	3
*CFI*	1
*C3*	1
Anti-FH autoantibody testing	
anti-FH autoantibody	1 (5)

The one recipient with anti-FH autoantibodies detected also had a variant of uncertain significance.

Of the nine recipients with variants in complement pathway genes, three had an existing diagnosis of aHUS (Supplementary dataset). No variants in non-complement genes associated with aHUS were identified in the genes that were tested.

### Current Transplant History

Details of the kidney transplants and recipients treated with reactive eculizumab are given in Table 3 including immunosuppression, where available.

**TABLE 3:** Details of kidney transplant treated for post-transplant TMA with eculizumab. SD, standard deviation.

Transplant details	Study cohort n = 26
Age at transplantation (mean ± SD)	40.8 ± 14.5 years
Live donor transplant (%)	13 (50)
First kidney transplant (%)	17 (65)
Mismatch (mean ± SD)	1.8 ± 1.4, n = 18
ABO incompatible transplant	2
**Induction immunosuppression**	
Basilixumab	8
Alemtuzumab	6
Data not available	12
**Maintenance immunosuppression**	
Tacrolimus + Mycophenolate mofetil + Prednisolone	17
Tacrolimus + Mycophenolate mofetil	1
Ciclosporin + Mycophenolate mofetil	1
Data not available	7

No transplant recipients received plasma exchange before transplantation with the aim of preventing aHUS recurrence. However, two (#592, #53, Supplementary dataset) had plasma exchange pre-transplant for desensitization in combination with rituximab.

### Presentation With Post-transplant TMA

#### Timing

**FIGURE 1:**
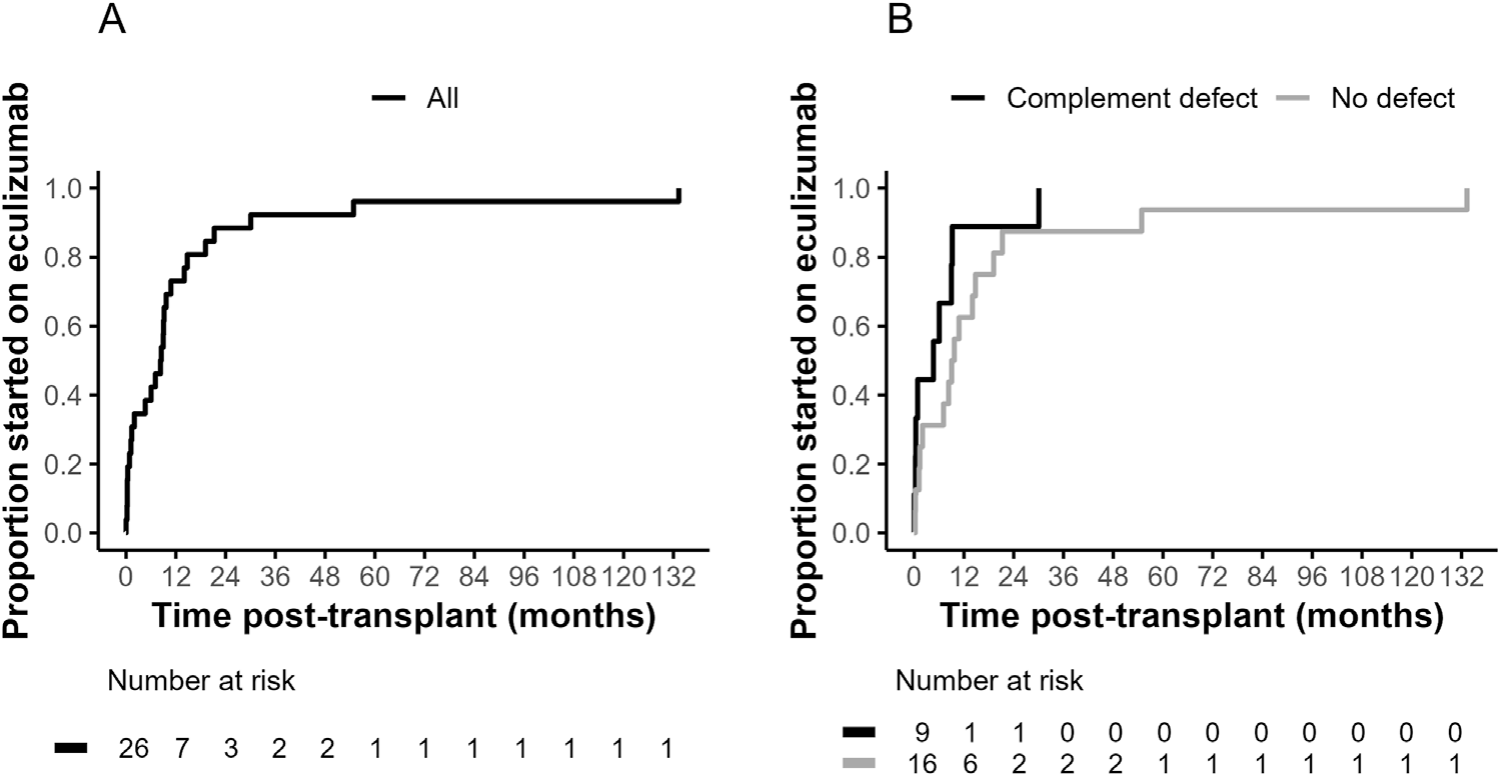
Kaplan-Meier curves for time post-transplant to initiation of eculizumab for treatment of post-transplant thrombotic microangiopathy. Data is shown for cohort as a whole **(A)** and grouped by presence of complement pathway defects **(B)** for those tested (log-rank *P* = 0.12).

Time from transplant to initiation of eculizumab for post-transplant TMA is shown in Figure 1A for all recipients in the cohort and grouped by presence of complement pathway defects (Figure 1B). Median (range) time at treatment was 8.4 months (6 h–11 years) post-transplant. Median time to presentation was under 12 months both for those with (4.5 months, range 6 h to 2.5 years) or without (9.0 months, range 7 days–11 years) complement pathway defects.

#### Renal Presentation of Post-transplant TMA

Five recipients (19.2%) required dialysis at the point of referral for post-transplant TMA for either acute kidney injury (AKI, n = 4) or delayed graft function (n = 1). Other renal presentations included AKI not requiring dialysis (n = 9), an acute rise in creatinine not meeting AKI criteria for magnitude (n = 5) [30], failure to achieve expected function of kidney transplant (n = 3) and non-acute progressive renal impairment (n = 4).

Patients who were biopsied (n = 24) had histological evidence of post-transplant TMA. Chronic damage was graded [31], where possible, from the available details in biopsy reports and is detailed in the Supplementary dataset with the majority graded as minimal or mild (n = 15). Timing of onset did not vary with degree of chronic damage (Supplementary Figure S1). The one adult recipient (#562) who did not have a transplant biopsy had hypertension as the presumed cause of native ESKD and a VUS in *C3*. She presented 9 days after transplantation of a well-matched live donor kidney, hypertensive with evidence of MAHA and a rising creatinine. The child (#4) who was not biopsied at the time of recurrence was treated as she had deteriorated within hours of transplant with concerns of MAHA including an elevated serum lactate dehydrogenase (LDH; 3334 IU/L), known aHUS and previous primary non function of first transplant.

#### Hematological Features at Presentation

Fifteen recipients (57.7%) had evidence of MAHA on presentation with post-transplant TMA with 13 having concurrent thrombocytopenia. A further three (11.5%) had isolated thrombocytopenia with the remainder having neither thrombocytopenia nor evidence of MAHA (n = 8, 30.8%). There was no difference in presence of hematological features by complement pathway defect status (Fisher exact test *P* = 1.0).

Five of the 18 (27.8%) with hematological features of TMA required dialysis at presentation compared to none of the 8 without hematological features of TMA (*P* = 0.281, Supplementary Table S2). There was no difference in time to onset of post-transplant TMA by hematological features (*P* = 0.405, Supplementary Table S2).

Of those presenting with MAHA (n = 15), LDH levels were available for 12 (Supplementary dataset). Median (range) serum LDH concentration in this group was 1,050 (222–3,338) IU/L. Data on LDH levels was not available for presentations without MAHA.

#### Evidence of Rejection

Two had evidence of peritubular capillary C4d staining on transplant biopsy at the time of post-transplant TMA occurrence. In one recipient (#593) with a pathological variant in *CFI* and previous transplant loss from arterial thrombus, peritubular capillary C4d staining was minimal and not diagnostic of AMR. Although no DSAs were detected, she was treated with methylprednisolone and received a trial of eculizumab for 2 weeks. Eculizumab was stopped due to a lack of improvement in renal function and the transplant failed shortly after at 5 months post-transplant. Graft loss was attributed to TMA rather than rejection. Cause of ESKD was listed as HIV nephropathy but was not biopsy confirmed.

In the other recipient (#591) peritubular capillary C4d staining was equivocal but associated with mild-moderate capillaritis. She was highly sensitized (98% panel reactivity at transplantation) and had existing low level DSAs to MHC class II antigens at transplantation. Eculizumab was authorised as a VUS in *CFI* was identified. Post-transplant TMA occurred 5 months post-transplant and a course of eculizumab was stopped after 2 months when AMR was thought more likely. Graft loss occurred 8 months later (15 months post-transplant) and was attributed to chronic AMR.

Four other recipients had glomerulitis and peritubular capillaritis (n = 3) or borderline changes suspicious for acute T cell mediated rejection on biopsy (n = 1). None had DSAs or peritubular capillary C4d staining.

Four had a history of DSAs but no biopsy features of rejection. Only two (#53, #589) had DSAs reported (against DQ6 and Cw6 respectively) at the point of post-transplant TMA occurrence and both were low levels.

### Other Treatments for Post-Transplant TMA

#### Plasma Exchange

Of those with available data (n = 24), 58.3% received plasma exchange at the time of post-transplant TMA occurrence (Supplementary dataset).

#### Change in CNI

Of the 25 recipients with available data, all were taking tacrolimus at the time of post-transplant TMA. Eight had their tacrolimus reduced or suspended initially with 2 subsequently reintroducing it. Belatacept was started in place of tacrolimus in four with a further one starting after an initial switch to sirolimus. Three did not have adjustments to their tacrolimus and one switched to ciclosporin. There was no data on adjustments to CNI immunosuppression for eight recipients.

### Outcomes

At last follow-up, 3 (11.5%) recipients had died with a functioning graft, 11 (42.3%) had grafts that continued to function and 12 (46.2%) had failed. In those surviving transplant recipients with a functioning graft, median (range) follow-up was 4.1 years (1.6–11.8 years) from initiation of eculizumab for post-transplant TMA.

#### Deaths

Two transplant recipients died with a functioning graft within the first year of transplantation. One (#566) died of a respiratory infection 1 month after starting eculizumab and 10 months after transplantation. This was his first transplant and native ESKD occurred in the context of severe hypertension and sepsis. The second (#577) died from a subarachnoid hemorrhage 5 months post-transplant. She had started eculizumab in the second month post-transplant and continued for 7 weeks until CNI toxicity was thought the more likely cause of post-transplant TMA. Tacrolimus had been stopped.

The third death with a functioning transplant was from an intracranial hemorrhage that occurred 10.3 years post-transplant and after almost 8 years of eculizumab treatment. This was the recipient’s (#273) third kidney transplant.

No deaths were recorded after graft failure (follow-up 3–59 months, median 19 months).

#### Graft Survival From Eculizumab Initiation

**FIGURE 2:**
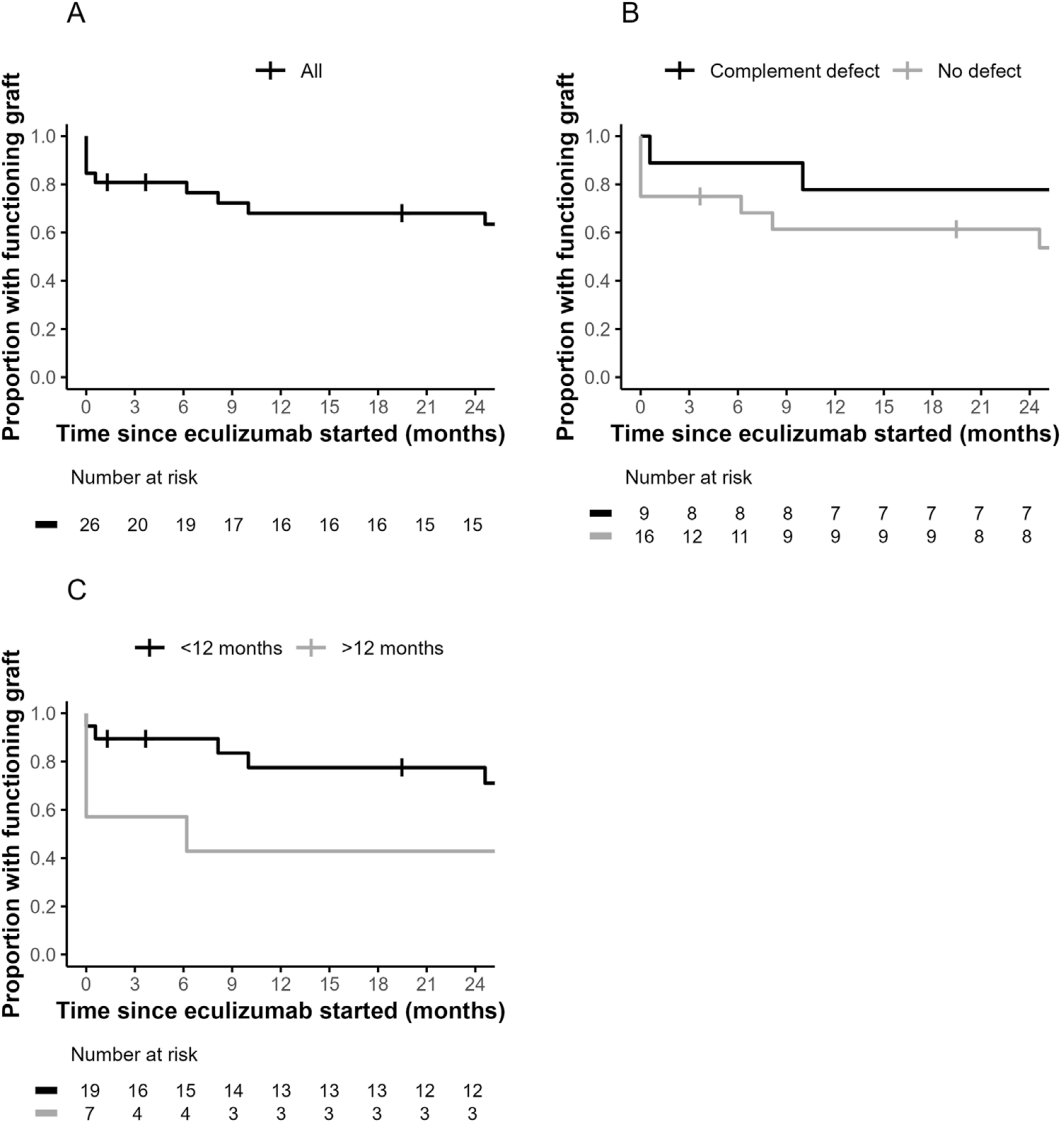
Death-censored Kaplan-Meier analysis of kidney graft survival from time of eculizumab initiation to treat post-transplant thrombotic microangiopathy. Data is shown for cohort as a whole **(A)**, grouped by presence of complement pathway defects (pathological variants or variants of uncertain significance in complement pathway genes or presence of autoantibodies against factor H) in those tested (log-rank *P* = 0.23) **(B)** or grouped by timing of eculizumab initiation in the post-transplant period (log-rank *P* = 0.36) **(C)**. Numbers at risk in each group at 3 monthly time points are detailed below the graph.

Death-censored kidney graft survival from the point of starting eculizumab for post-transplant TMA is shown in Figure 2A. One year after starting eculizumab, death-censored transplant survival was 68.0%.

Death-censored graft survival was 70.7% for those with and 65.5% for those without complement pathway defects, 1 year after starting eculizumab treatment (Figure 2B). Amongst those who presented within 12 months of transplant, there was no clear difference in outcome between those with or without complement pathway defects (Supplementary Figure S2). Graft outcomes were similar in those with or without hematological features of TMA at presentation (Supplementary Figure S3) and between grades of chronic damage on biopsy (Supplementary Figure S4).

There is a suggestion that later presentation with post-transplant TMA is associated with worse kidney graft survival subsequently but this difference did not reach statistical significance (*P* = 0.36). Death-censored graft survival 12 months after eculizumab initiation was 42.9% for those starting after the first year of transplantation, compared to 77.5% for those starting earlier (Figure 2C). There was no difference in proportion with complement pathway defects, hematological features of TMA or existing diagnoses of aHUS between those presenting early or late (Supplementary Table S3). Those presenting later more commonly required dialysis at presentation (*P* = 0.01, Supplementary Table S3).

In the 6 recipients with pathogenic variants in complement pathway genes or anti-FH autoantibodies, 5 presented within 12 months of transplant including 3 grafts that failed during follow-up. One graft failed shortly after starting eculizumab whereas the other two continued to function over 4 years into eculizumab treatment. For those with pathogenic variants or anti-FH autoantibodies, graft survival appears similar to prophylactic eculizumab treatment (Supplementary Figure S5) [1, 14].

#### Outcomes in Those on Dialysis at Presentation

Of the 5 recipients on dialysis at the point of referral for post-transplant TMA, all those (n = 4) presenting more than 12 months post-transplant either remained dialysis dependent (n = 3) or experienced graft loss 6 months after starting eculizumab (n = 1). In the other case, post-transplant TMA occurred early in the post-transplant period and the graft continued to function 3 years later.

#### Cause of Graft Loss

Graft loss was attributed to either TMA (n = 10) or rejection (n = 2). One case (#591) attributed to rejection is discussed above and eculizumab was stopped before graft loss. In the second case (#99) graft loss occurred 5.2 years into eculizumab treatment which was started 12 days after transplantation.

Graft loss from TMA mostly occurred within 12 months of starting eculizumab treatment. In five cases transplant loss was less than 1 month after presentation, in two cases it occurred six to 8 months later and in a further three cases the graft functioned for at least 2 years from starting eculizumab.

#### TMA Recurrence on Eculizumab Treatment

There were no reported episodes of clinical TMA recurrence in transplants included in this cohort, after the episode treated with eculizumab.

One recipient (#590) was biopsied for worsening proteinuria 5 months after stopping eculizumab and this showed evidence of acute TMA and damage from previous TMA. No further eculizumab was given as the native kidney disease had been reassessed as not being complement mediated. The graft continued to function at 3 years.

#### Cessation of Eculizumab

Eleven (42.3%) stopped complement blockade at a point when their graft was still functioning. In two the rationale was failure for graft function to improve and both grafts were lost to TMA shortly after. In three cases it was a local team decision or patient preference to stop eculizumab treatment after at least six completed months. Two had no identified complement pathway defects and the third had a VUS in *CFI*. Follow-up in these three recipients is at least 2 years from treatment cessation, with no reported TMA recurrence.

In the remaining six transplant recipients, eculizumab treatment was stopped when TMA was thought more likely secondary to an alternative diagnosis. This was most commonly CNI toxicity but also included possible IgA recurrence and AMR. In all these cases, eculizumab was stopped within 6 months of initiation. One recipient died a few months later from unrelated causes (#577) and another (#591) lost their graft to rejection 8 months after cessation. The remaining 4 had at least 18 months follow-up from stopping eculizumab and no reported clinical TMA recurrence, although one (#590) discussed above had TMA on repeat biopsy.

## Discussion

We present our experience of treating 26 selected cases of post-transplant TMA with eculizumab, coordinated through a national specialist centre. Our cohort adds to the existing data on eculizumab treatment for post-transplant TMA by including both *de novo* presentations and those with an existing diagnosis of aHUS, providing details on time to presentation, presence of inherited and acquired alternative complement pathway defects and rationale for treatment in those presenting without an existing diagnosis of aHUS. Follow-up extended to at least 18 months from eculizumab initiation for those whose grafts continued to function.

Perhaps the most comparable cohort to our dataset is the CUREiHUS cohort of 15 transplant recipients treated for post-transplant TMA with eculizumab, of which a third had no previous history of aHUS [13]. Despite the inclusion of *de novo* post-transplant TMA 60% were found to have a variant in a complement pathway gene. Only three (20%) grafts failed during the study period but three (25%) of the remaining grafts had follow-up of less than 4 months from eculizumab initiation which is short relative to what we present.

Genetic testing forms an integral part of diagnosing aHUS and it is generally reported that 50%–70% of people with aHUS have a genetic variant or autoantibody causing complement pathway dysregulation [1, 2, 8–10, 32, 33]. However, in our cohort the decision to treat with eculizumab was often made in advance of these results, given the time sensitivity of treatment initiation [8]. Ultimately, only 34.6% of the recipients had an identified complement pathway defect. The heterogeneity of our cohort likely explains the relatively low detection of complement pathways defects as only three (11.5%) had an existing diagnosis of aHUS. In support of this, others have found a low rate of 20% with complement pathway defects in recipients with *de novo* post-transplant disease [2].

Post-transplant TMA is not always associated with hematological features of thrombocytopenia and MAHA but can instead be limited to kidney involvement identified on biopsy [4, 34]. Some trials using eculizumab post-transplant excluded those without hematological features of TMA [35], this was not the case for our cohort where 30.7% did not have hematological evidence of TMA. This is similar to previously reported rates in a cohort of 21 cases of *de novo* post-transplant TMA where 38% had only kidney involvement [34]. Schwimmer et al. [34] found that those with hematological evidence of TMA presented earlier and were more likely to require dialysis. Amongst our cohort there was no difference in timing of TMA onset by hematological status and the higher requirement for dialysis was not statistically significant. The presence of complement pathway defects between those with and without hematological features did not differ either.

A key finding in our cohort is apparent poor graft survival following post-transplant TMA, despite eculizumab treatment, particularly for those presenting with post-transplant TMA >12 months after transplantation. Delayed recognition may worsen outcomes for those presenting later when monitoring is less frequent. Disease in transplanted kidneys can also take longer to respond to eculizumab compared to native kidneys [4], highlighting the need for prompt treatment. Supporting this hypothesis of more severe kidney injury with later presentations, more of those presenting >12 months post-transplant required dialysis (Supplementary Table S3). Alternatively, worse outcomes for later presentations may represent more non-complement mediated TMA, not expected to respond to eculizumab. aHUS recurrence after transplantation typically occurs early [1], although late presentations are documented [12, 14, 36]. Our cohort is a heterogenous one but there was no clear split in terms of existing and *de novo* disease or complement pathway defects between those presenting before or after 1 year of transplantation.

Complement mediated HUS is a rare cause of post-transplant TMA that requires prompt recognition to be appropriately treated. Genetic testing is an important element in the diagnostics but these results can take some time. Genetic testing can also support clinicians in revisiting the listed diagnosis of ESKD if variants are identified, particularly if historic native kidney biopsies were ambiguous. Indeed, in four (15.4%) of the cases presented here, complement pathway defects were identified after referral for post-transplant TMA raising the suspicion of aHUS contributing to native ESKD. It is essential to differentiate between hypertensive and complement mediated disease before transplant to better guide post-transplant care and genetic testing can contribute to this [11, 16].

We suggest screening for complement defects prior to transplantation to allow access to prophylactic eculizumab if a medium or high-risk variant is identified in selected patients: with ESKD due to Shiga toxin-producing *E. coli* HUS, when the cause of ESKD is unknown but associated with hematological features of TMA or accelerated hypertension or if there has been a TMA in a previous transplant. In those presenting post-transplant with a TMA, continuation of reactive eculizumab treatment should be reassessed early in those presenting after the first year of transplantation given poor graft outcomes.

## Data Availability

The original contributions presented in the study are included in the article/Supplementary Material, further inquiries can be directed to the corresponding author.
