# Supplementary material for: Late Onset Thrombotic Microangiopathy in Kidney Transplants; Poor Outcome Despite Eculizumab Treatment
Source: Transpl Int. 2025 Nov 24;38:15404. doi: 10.3389/ti.2025.15404 (PMC12684766; doi:10.3389/ti.2025.15404)
Supplement: Supplementary file 1 [file DataSheet1.pdf]

Supplementary material

Supplementary dataset.

Data for transplants where the recipients received eculizumab treatment from the point of post-transplant thrombotic microangiopathy (TMA) occurrence for presumed complement mediated TMA. Where a genetic variant in a gene of the complement pathway is identified, this is categorised as pathological or a variant of uncertain significance (VUS) and details of the variant are provided. Presence of antibodies to factor H (anti-FH) are given where tested. The combination of genetic and antibody testing is used to categorise recipients by whether they have an identified defect in the complement pathway or not. aHUS diagnosis is given as "Existing" for those with recognised native end stage kidney disease (ESKD) from aHUS before transplantation and "de novo" for those where the first recognised episode of probably complement mediated TMA occurred post-transplant. Graft survival is at time of last follow up or patient death if died with functioning graft. Kidney transplant biopsy result from the time of post-transplant TMA is given, including a grading of chronic damage (31) when available. Reference numbers (NCL#) are for individual transplant recipients. ABOi, ABO incompatible transplant; AMR, antibody mediated rejection; CAMR, chronic antibody mediated rejection; CFH, Complement Factor H; CFI, Complement Factor I; CNI, calcineurin inhibitor; DBD, donor after brainsteam death; DCD, donor after circulatory death; DSAs, donor specific antibodies; FSGS, focal segmental glomerulosclerosis; LD, living donor; MAHA, microangiopathic hemolytic anaemia; MMF, mycophenolate mofetil; MPGN, membranoproliferative glomerulonephritis; PLEX, plasma exchange; Pred, prednisolone; Tac, Tacrolimus; TCMR, T cell mediated rejection; Tx, transplant.

Supplementary Figure 1. Kaplan-Meier curves for time post-transplant to initiation of eculizumab for treatment of post-transplant thrombotic microangiopathy. Subjects are grouped by grade of chronic damage (minimal, mild or moderate) of kidney transplant biopsy (log-rank P = 0.53). Numbers at risk in each group at 3 monthly time points are detailed below the graph. Median (interquartile range) time to onset was similar between the groups at 207 (51-328), 212 (111-252) and 575 (423-739) days post-transplant in those with minimal, mild or moderate chronic damage, respectively (Kruskal-Wallis P = 0.225).

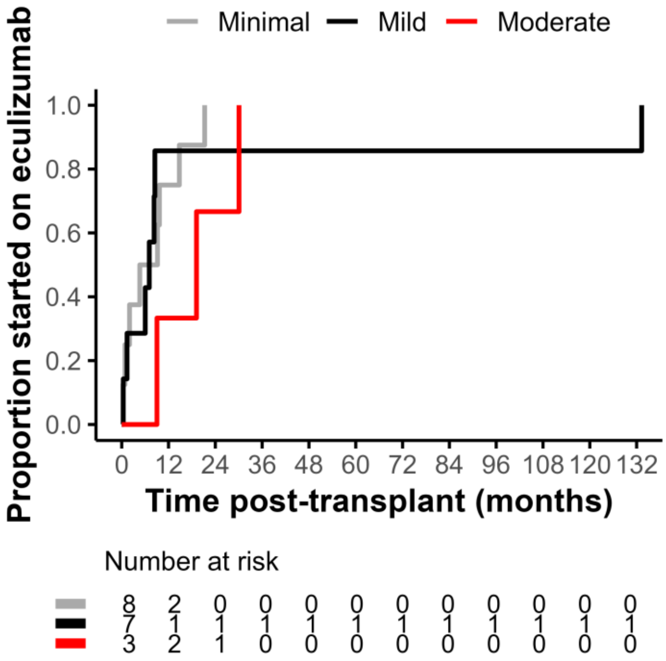

Supplementary Figure 2. Death-censored Kaplan-Meier analysis of kidney graft survival from time of eculizumab initiation to treat post-transplant thrombotic microangiopathy in those recipients who started eculizumab treatment within the first year of transplantation. Subjects are grouped by presence of complement pathway defects. Numbers at risk in each group at 3 monthly time points are detailed below the graph. Log-rank *P* = 0.29.

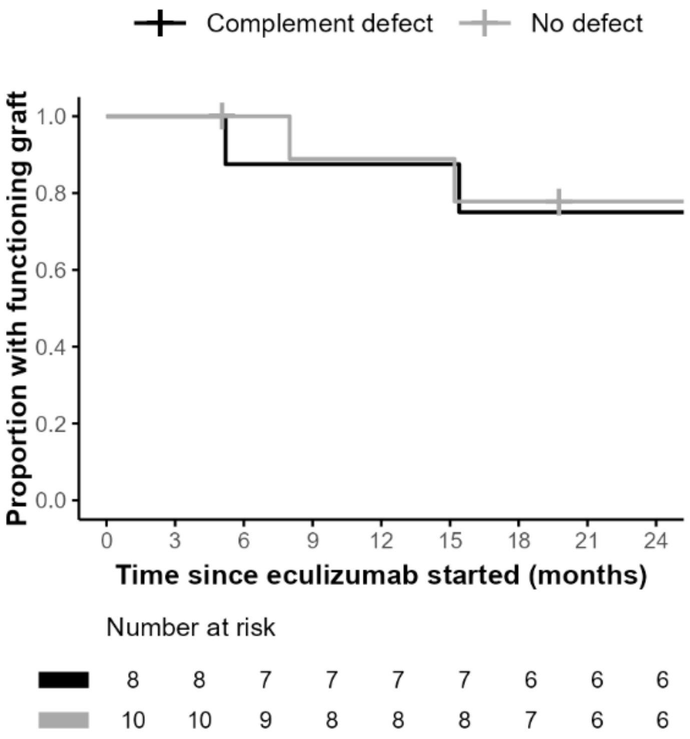

Supplementary Figure 3. Death-censored Kaplan-Meier analysis of kidney graft survival from time of eculizumab initiation to treat post-transplant thrombotic microangiopathy (TMA). Subjects are grouped by presence or absence of hematological features of TMA, namely microangiopathic anaemia with or without concurrent thrombocytopenia (MAHA) or isolated thrombocytopenia. In **(A)** subjects with MAHA and isolated thrombocytopenia are grouped together (log-rank $P = 0.44$) whereas in **(B)** those with isolated thrombocytopenia (n=3) have been excluded from analysis (log-rank $P = 0.36$). Numbers at risk in each group at 3 monthly time points are detailed below the graph.

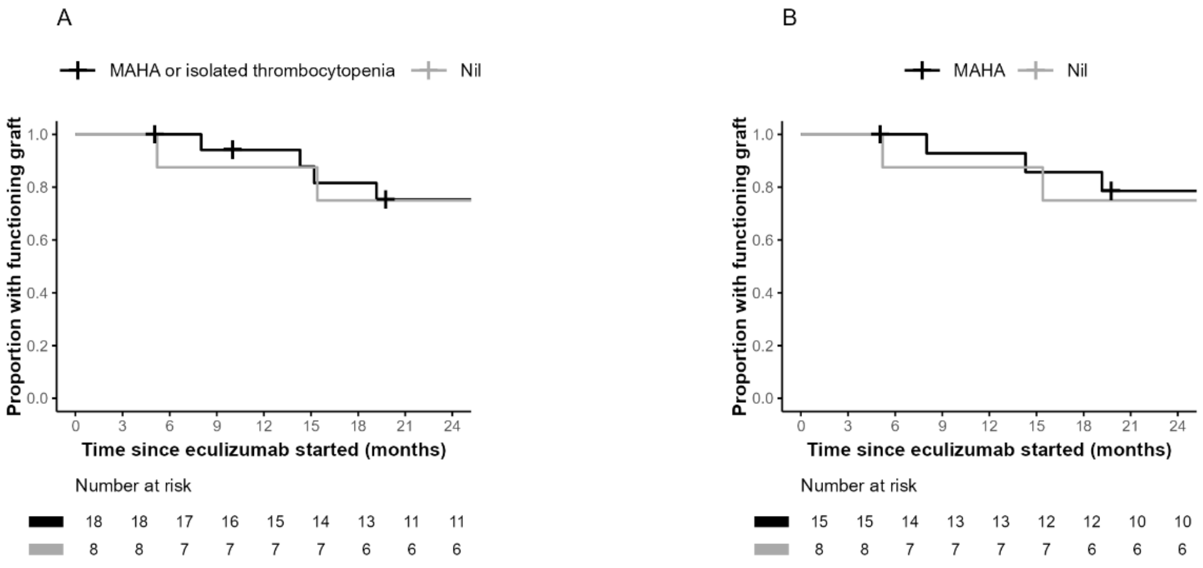

Supplementary Figure 4. Death-censored Kaplan-Meier analysis of kidney graft survival from time of eculizumab initiation to treat post-transplant thrombotic microangiopathy. Subjects are grouped by grade of chronic damage (minimal, mild or moderate) on kidney transplant biopsy (log-rank $P = 0.21$). Numbers at risk in each group at 3 monthly time points are detailed below the graph.

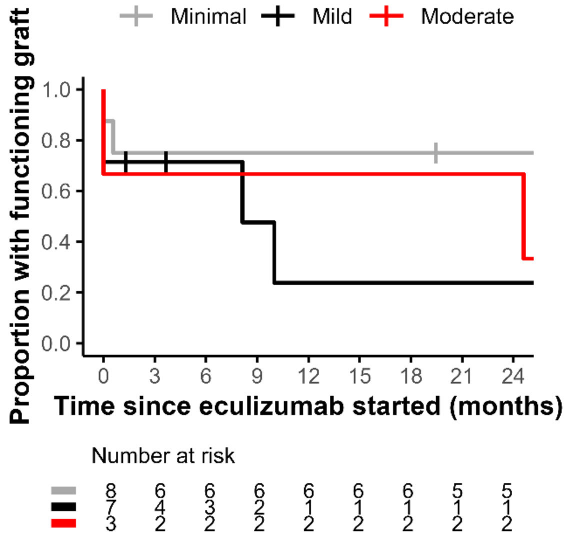

Supplementary Figure 5. Death-censored Kaplan-Meier analysis of kidney graft survival in those with pathogenic complement factor H variants or factor H autoantibodies treated with either prophylactic eculizumab (black) from the time of transplantation, or reactive eculizumab (grey) at the point of post-transplant thrombotic microangiopathy occurrence. Number at risk in each group at 12 monthly time points are detailed below the graph. The prophylactic eculizumab group are those with the above complement defects within the previously published UK cohort.(1) Log-rank *P* =0.35.

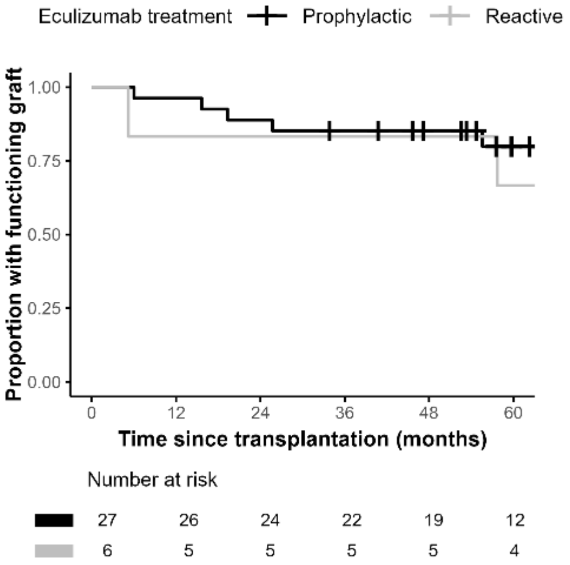

Supplementary Table 1. Criteria advised by KDIGO to determine level of risk that atypical hemolytic uremic syndrome (aHUS) will recur in transplant kidney.(1, 9)

**High-risk**	**Medium-risk**	**Low-risk**
Mutations in Factor H or gene rearrangements involving Factor H or factor H related proteins<br>Gain of function mutations in Factor B or C3<br>Loss of previous transplant due to recurrent aHUS	No identified mutation or autoantibody<br>Mutations in Factor I<br>Mutations of uncertain functional significance<br>Detectable autoantibodies against Factor H	Mutation in membrane cofactor protein CD46<br>Previous autoantibody positivity

Supplementary Table 2. Comparison of features in those presenting with or without hematological features (microangiopathic hemolytic anaemia and/or thrombocytopenia) of thrombotic microangiopathy. Variant of uncertain significance (VUS). P values for characteristics comparisons were calculated with Fisher exact test for categorical variables, t-test to compare means and Kruskal-Wallis to compare medians. AKI, acute kidney injury; IQR, interquartile range; SD, standard deviation

		Without	With	Total	p
**Total N (%)**		8 (30.8)	18 (69.2)	26	
**Onset of eculizumab treatment in post-transplant period**	Early (<12 months post-transplant)	6 (75.0)	13 (72.2)	19 (73.1)	1
	Late (>12 months post-transplant)	2 (25.0)	5 (27.8)	7 (26.9)	
**Onset of eculizumab treatment in days post-transplant**	Median (IQR)	270.0 (170.5 to 348.2)	230.5 (15.2 to 404.8)	252.0 (36.5 to 396.8)	0.405
**Recipient sex**	Female	4 (50.0)	15 (83.3)	19 (73.1)	0.149
	Male	4 (50.0)	3 (16.7)	7 (26.9)	
**Recipient age at transplantation (years)**	Mean (SD)	38.5 (12.0)	41.8 (16.1)	40.8 (14.8)	0.564
**Identified complement pathway defects**	None	5 (62.5)	11 (61.1)	16 (61.5)	1
	Pathological	2 (25.0)	3 (16.7)	5 (19.2)	
	VUS	1 (12.5)	3 (16.7)	4 (15.4)	
	Not tested		1 (5.6)	1 (3.8)	
**Diagnosis**	de novo	8 (100.0)	15 (83.3)	23 (88.5)	0.529
	Existing		3 (16.7)	3 (11.5)	
**Donor type**	Live	5 (62.5)	8 (44.4)	13 (50.0)	0.673
	Deceased	3 (37.5)	10 (55.6)	13 (50.0)	
**Mismatch**	Mean (SD)	2.7 (1.6)	1.4 (1.2)	1.8 (1.4)	0.134
**Requiring dialysis at referral**	No	8 (100.0)	13 (72.2)	21 (80.8)	0.281
	Yes		5 (27.8)	5 (19.2)	
**Renal presentation at time of post-transplant TMA**	AKI	3 (37.5)	10 (55.6)	13 (50.0)	0.854
	Delayed graft function		1 (5.6)	1 (3.8)	
	Failure to achieve expected function	1 (12.5)	2 (11.1)	3 (11.5)	
	Progressive renal impairment	2 (25.0)	2 (11.1)	4 (15.4)	
	Rising creatinine	2 (25.0)	3 (16.7)	5 (19.2)	

Supplementary Table 3. Comparison of features in those presenting with post-transplant thrombotic microangiopathy in the early (before 12 months post-transplant) or late (after 12 months of transplantation) post-transplant period. Variant of uncertain significance (VUS). P values for characteristics comparisons were calculated with Fisher exact test for categorical variables, t-test to compare means. AKI, acute kidney injury; SD, standard deviation

		**Early**	**Late**	**Total**	**p**
**Total N (%)**		19 (73.1)	7 (26.9)	26	
**Recipient sex**	Female	14 (73.7)	5 (71.4)	19 (73.1)	1
	Male	5 (26.3)	2 (28.6)	7 (26.9)	
**Recipient age at transplantation (years)**	Mean (SD)	41.7 (14.9)	38.3 (15.2)	40.8 (14.8)	0.621
**Identified complement pathway defects**	None	10 (52.6)	6 (85.7)	16 (61.5)	0.607
	Pathological	4 (21.1)	1 (14.3)	5 (19.2)	
	VUS	4 (21.1)		4 (15.4)	
	Not tested	1 (5.3)		1 (3.8)	
**Diagnosis**	de novo	17 (89.5)	6 (85.7)	23 (88.5)	1
	Existing	2 (10.5)	1 (14.3)	3 (11.5)	
**Donor type**	Live	8 (42.1)	5 (71.4)	13 (50.0)	0.378
	Deceased	11 (57.9)	2 (28.6)	13 (50.0)	
**Mismatch**	Mean (SD)	2.0 (1.4)	1.0 (1.7)	1.8 (1.4)	0.427
**Evidence of hematological features of TMA**	No	6 (31.6)	2 (28.6)	8 (30.8)	1
	Yes	13 (68.4)	5 (71.4)	18 (69.2)	
**Requiring dialysis at referral**	No	18 (94.7)	3 (42.9)	21 (80.8)	0.01
	Yes	1 (5.3)	4 (57.1)	5 (19.2)	
**Renal presentation at time of post-transplant TMA**	AKI	8 (42.1)	5 (71.4)	13 (50.0)	0.281
	Delayed graft function	1 (5.3)		1 (3.8)	
	Failure to achieve expected function	3 (15.8)		3 (11.5)	
	Progressive renal impairment	2 (10.5)	2 (28.6)	4 (15.4)	
	Rising creatinine	5 (26.3)		5 (19.2)	
